# Improving the genome and proteome annotations of the marine model diatom *Thalassiosira pseudonana* using a proteogenomics strategy

**DOI:** 10.1007/s42995-022-00161-y

**Published:** 2023-02-03

**Authors:** Xiao-Huang Chen, Ming-Kun Yang, Yuan-Yuan Li, Zhang-Xian Xie, Shu-Feng Zhang, Mats Töpel, Shady A. Amin, Lin Lin, Feng Ge, Da-Zhi Wang

**Affiliations:** 1 State Key Laboratory of Marine Environmental Science/College of the Environment and Ecology, Xiamen University, Xiamen, 361005 China; 2 Southern Marine Science and Engineering Guangdong Laboratory (Zhuhai), Sun Yat-Sen University, Zhuhai, 519082 China; 3 Key Laboratory of Algal Biology, Institute of Hydrobiology, Chinese Academy of Sciences, Wuhan, 430072 China; 4 College of Chemistry, Chemical Engineering and Environment, Minnan Normal University, Zhangzhou, 363000 China; 5 Department of Marine Sciences, University of Gothenburg, Box 461, 40530 Gothenburg, Sweden; 6 IVL-Swedish Environmental Research Institute, Box 53021, 40014 Gothenburg, Sweden; 7 New York University Abu Dhabi, Saadiyat Island, 129188 Abu Dhabi, United Arab Emirates

**Keywords:** Marine diatoms, *Thalassiosira pseudonana*, Proteome, Proteogenomics

## Abstract

Diatoms are unicellular eukaryotic phytoplankton that account for approximately 20% of global carbon fixation and 40% of marine primary productivity; thus, they are essential for global carbon biogeochemical cycling and climate. The availability of ten diatom genome sequences has facilitated evolutionary, biological and ecological research over the past decade; however, a complimentary map of the diatom proteome with direct measurements of proteins and peptides is still lacking. Here, we present a proteome map of the model marine diatom *Thalassiosira pseudonana* using high-resolution mass spectrometry combined with a proteogenomic strategy. In-depth proteomic profiling of three different growth phases and three nutrient-deficient samples identified 9526 proteins, accounting for ~ 81% of the predicted protein-coding genes. Proteogenomic analysis identified 1235 novel genes, 975 revised genes, 104 splice variants and 234 single amino acid variants. Furthermore, our quantitative proteomic analysis experimentally demonstrated that a considerable number of novel genes were differentially translated under different nutrient conditions. These findings substantially improve the genome annotation of *T. pseudonana* and provide insights into new biological functions of diatoms. This relatively comprehensive diatom proteome catalog will complement available diatom genome and transcriptome data to advance biological and ecological research of marine diatoms.

**Supplementary Information:**

The online version contains supplementary material available at 10.1007/s42995-022-00161-y.

## Introduction

Diatoms are one of the most common, diverse and ecologically important phytoplankton groups in the ocean (Malviya et al. 2016). They are responsible for approximately 20% of global carbon fixation and 40% of marine primary productivity (Nelson et al. 1995) and form a substantial component of the coastal marine food web. Furthermore, diatom-dominated spring phytoplankton blooms are also considered to be a crucial part of the marine biological pump (Martina et al. 2011; Turner 2002). Thus, marine diatoms regulate the biogeochemical cycles of many biogenic elements (such as carbon, nitrogen, phosphorus and silicon) and global climate (Bopp et al. 2005; Föllmi 1996; Smetacek 1999; Tréguer et al. 1995; Voss et al. 2013). Additionally, diatoms provide a perspective for the production of drugs, biofuels, biomedicine and nanostructured materials (Dolatabadi and de la Guardia 2011; Hu et al. 2008; Ragni et al. 2018).

In the past 10 to 20 years, the genomes of ten diatom species have been sequenced, revealing many novel metabolic pathways for this organism group, such as C4 photosynthesis and the urea cycle and providing novel insights into evolution, biology and ecology of diatoms (Armbrust et al. 2004; Basu et al. 2017; Bhattacharjya et al. 2021; Bowler et al. 2008; Galachyants et al. 2019; Lommer et al. 2012; Mock et al. 2017; Ogura et al. 2018; Osuna-Cruz et al. 2020; Tanaka et al. 2015). However, an equivalent map for the diatom proteome with direct measurements of proteins and peptides is lacking, due to the limitation of available technology and lack of high-quality genome annotation. To date, a large proportion of diatom genes are lacking a functional annotation and the majority of predicted protein-coding genes have not been validated at the functional level. Recent advancements in mass spectrometry (MS) technology have resulted in the rapid development of proteomics. Several studies have cataloged daft proteome maps from unicellular organisms to higher organisms, from subcellular structures to tissues, using deep and complete proteomic analysis (Christoforou et al. 2016; Kim et al. 2014; Schober et al. 2019; Thul et al. 2017; Wilhelm et al. 2014; Yang et al. 2014, 2018).

Current proteomic methods face challenges in identifying proteins due to their dependence on predefined protein sequence databases. To overcome this limitation, proteogenomics, an emerging field in which MS-acquired proteomic data are used to annotate genomes, has been developed (Jaffe et al. 2004) and successfully applied in a few model organisms (Kim et al. 2014; Ruggles et al. 2017; Wilhelm et al. 2014; Wright et al. 2016; Yang et al. 2014, 2018). These studies indicate that the proteogenomic pipeline combining the in-depth MS-based proteomic approach with high-throughput genomic and transcriptomic data significantly improves the genome annotations that benefits comprehensive studies of proteomics and expands our understanding of gene structures (Fermin et al. 2006; Jaffe et al. 2004; Nesvizhskii 2014).

The genome of the marine centric diatom *Thalassiosira pseudonana* was the first sequenced diatom genome and served as a model diatom (Armbrust et al. 2004; Oudot-Le Secq et al. 2007); it was subsequently improved in 2008 (Armbrust et al. 2008). The completed genome is about 32.4 Mb long and is estimated to contain 11,776 genes (Armbrust et al. 2008). However, more than half of these predicted genes are not yet functionally annotated, which impedes our comprehensive understanding of molecular and cellular processes, despite the many studies that have explored the ecological significance of diatoms (Chen et al. 2018; Dong et al. 2016; Dyhrman et al. 2012; Muhseen et al. 2015; Smith et al. 2016). Therefore, it is desirable to generate high-quality proteome data from *T. pseudonana* and thereby improve the genome annotation and facilitate further in-depth studies of diatoms. To that end, we have developed a proteome map of *T. pseudonana* by systematically identifying and annotating protein-coding genes in the diatom genome using high-resolution MS data derived from a sequential extraction and enrichment method. We have also applied a newly developed eukaryotic proteogenomic approach (Yang et al. 2018) based on high-quality proteomic data to improve the genomic annotation of *T. pseudonana*. Our study reveals novel protein-coding genes that are missing in the current version of the genome annotation, and the constructed proteome map provides a comprehensive protein database for direct characterization of metabolic activities under various environmental conditions, that will facilitate biological and ecological studies of marine diatoms.

## Results

### Generating a high-quality MS dataset

**Fig. 1:**
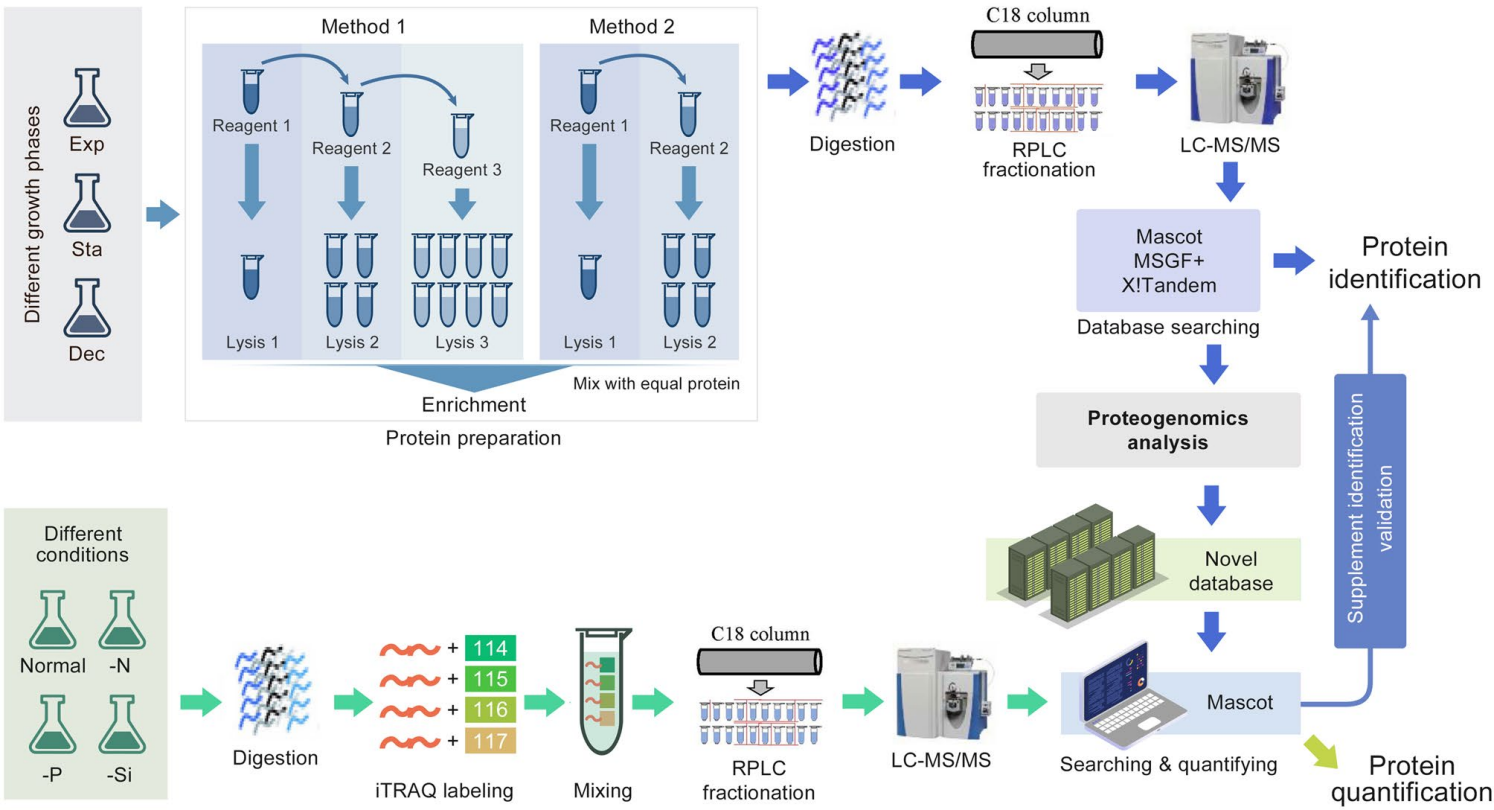
Workflow for protein identification of *Thalassiosira pseudonana*

To generate a baseline proteomic profile of *T. pseudonana*, three samples collected at the exponential, stationary and decline phases were subjected to proteomic analysis (Fig. 1). Proteins from each sample were sequentially extracted using two methods, each with multiple steps, and proteins were enriched at each step. After the fractionation with reversed-phase columns at the peptide level, the peptide mixtures were analyzed using a high-resolution and highly accurate mass spectrometer. Three different search engines, MSGF + , X!Tandem and Mascot were used to search the high-quality MS spectra against the predicted protein database to increase peptide identification (Supplementary Data Set 1). The identified peptides from the different growth phases and different search engine are shown in Supplementary Fig. S1A, B. In total, 52,804 high-confidence peptides corresponding to 508,560 spectra were identified after false discovery rate (FDR) filtering of no more than 1%. Among them, 51,006 unique peptides were used for protein identification.

After de-redundancy, a total of 11,727 protein-coding genes were annotated in the *T. pseudonana* reference genome, including 11,673 in the nuclear genome, 141 in the chloroplast genome, and 35 in the mitochondrial genome (Armbrust et al. 2004; Bowler et al. 2008; Oudot-Le Secq et al. 2007). In the present study, a total of 9526 predicted genes were identified (Supplementary Data Set 2), including 7339 identified by at least two unique peptides, 1888 identified by a single unique peptide with manual validation (Supplementary Fig. S1C), and 299 supplemented by our previous quantitative proteomic study (Chen et al. 2018). The genes identified by the MS data accounted for ~ 81% of the annotated protein-coding genes in the *T. pseudonana* reference genome. The median number of unique peptides identified per gene was six, whereas the median protein sequence coverage and the highest protein sequence coverage were ~ 12% and ~ 79%, respectively (Supplementary Fig. S1D, E). For example, the gene coding for the glyceraldehyde-3-phosphate dehydrogenase precursor, was identified by 75 peptides contributing protein sequence coverage of 75% (Supplementary Fig. S1E). Functional annotation of all predicted- and identified genes were analyzed by querying the Gene Ontology (GO), EuKaryotic Orthologous Groups (KOG) and Kyoto Encyclopedia of Genes and Genomes (KEGG) database (Supplementary Data Set 2 and Supplementary Fig. S2A). This analysis, which resulted in 7418 predicted genes, had a functional annotation either in GO, KOG or KEGG, accounting for ~ 63% of all predicted genes. A total of 6334 identified genes with functional annotation accounted for about 85% of predicted genes with functional annotation. Most of the identified proteins were predicted to be located in the nucleus (4149), cytoplasm (1953), plasma membrane (1284), chloroplast (966), extracellular areas (623), and mitochondria (519), and cytoplasmic proteins were the most easily detected as they accounted for the highest proportions (88%) of their predicted gene counterparts (Supplementary Fig. S2B).

**Fig. 2:**
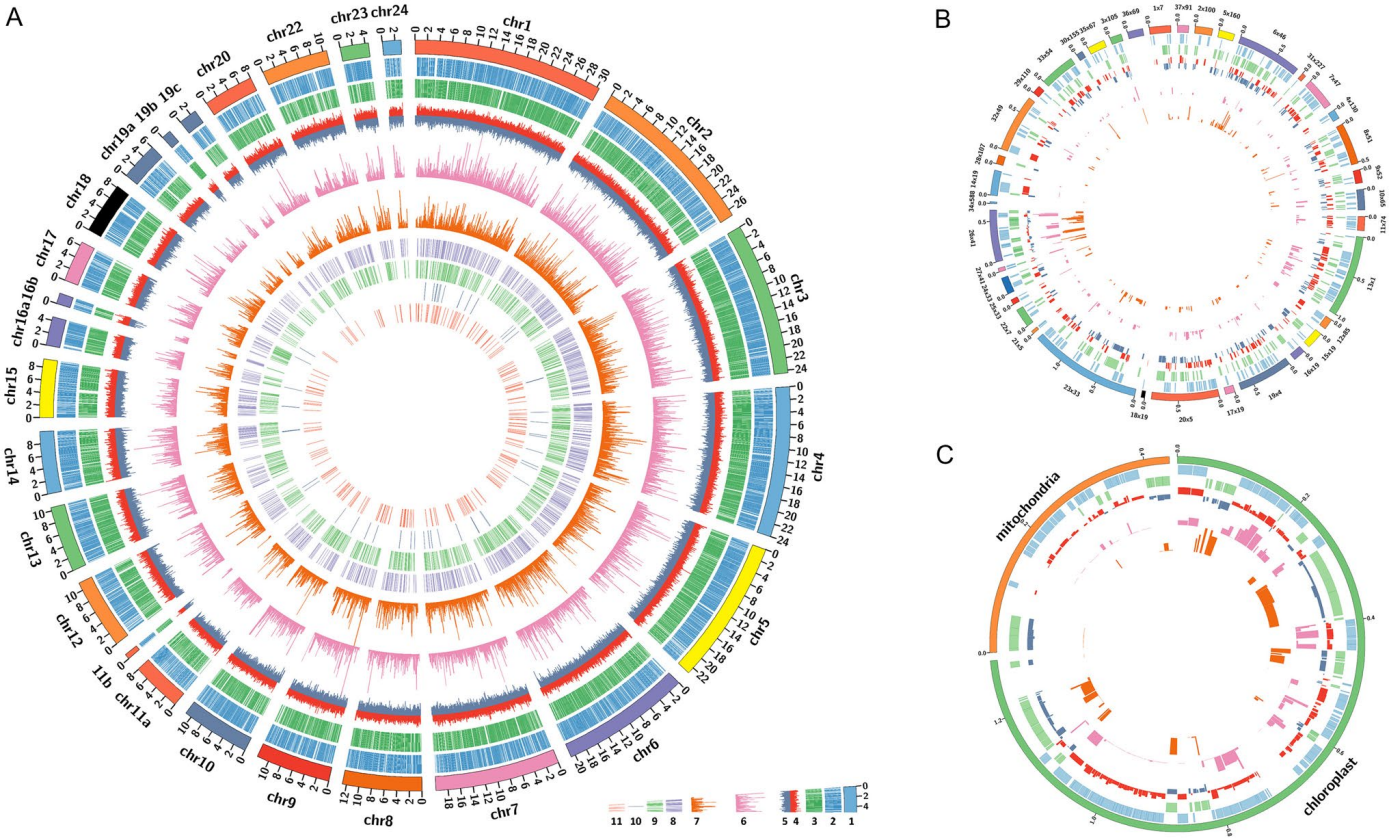
Overview of the proteomic and proteogenomic results. **A** Chromosomes; **B** Unmapped sequences; **C** Mitochondrial and chloroplast sequences. Circles: 1, chromosome; 2, predicted gene on the plus strand; 3, predicted gene on the minus strand; 4, GC content of predicted gene on the plus strand; 5, GC content of predicted gene on minus strand; 6, identified peptide sequence coverage of predicted gene on the plus strand; 7, identified peptide sequence coverage of predicted gene on the minus strand; 8, novel genes; 9, revised genes; 10, splice variants; 11, single amino acid variants

The distribution of predicted protein-coding genes and identified proteins on different chromosomes, unmapped sequence regions, and organelles, is shown in Fig. 2. A total of 9245 (82%) predicted protein-coding genes in the nuclear genome were confirmed to translate a product. Three hundred and seventy-seven protein-coding genes were located in unmapped sequence regions of gene model and 233 of them were identified. One hundred and ten protein-coding genes were identified in the chloroplast genome and 27 protein-coding genes were detected in the mitochondrial genome.

**Fig. 3:**
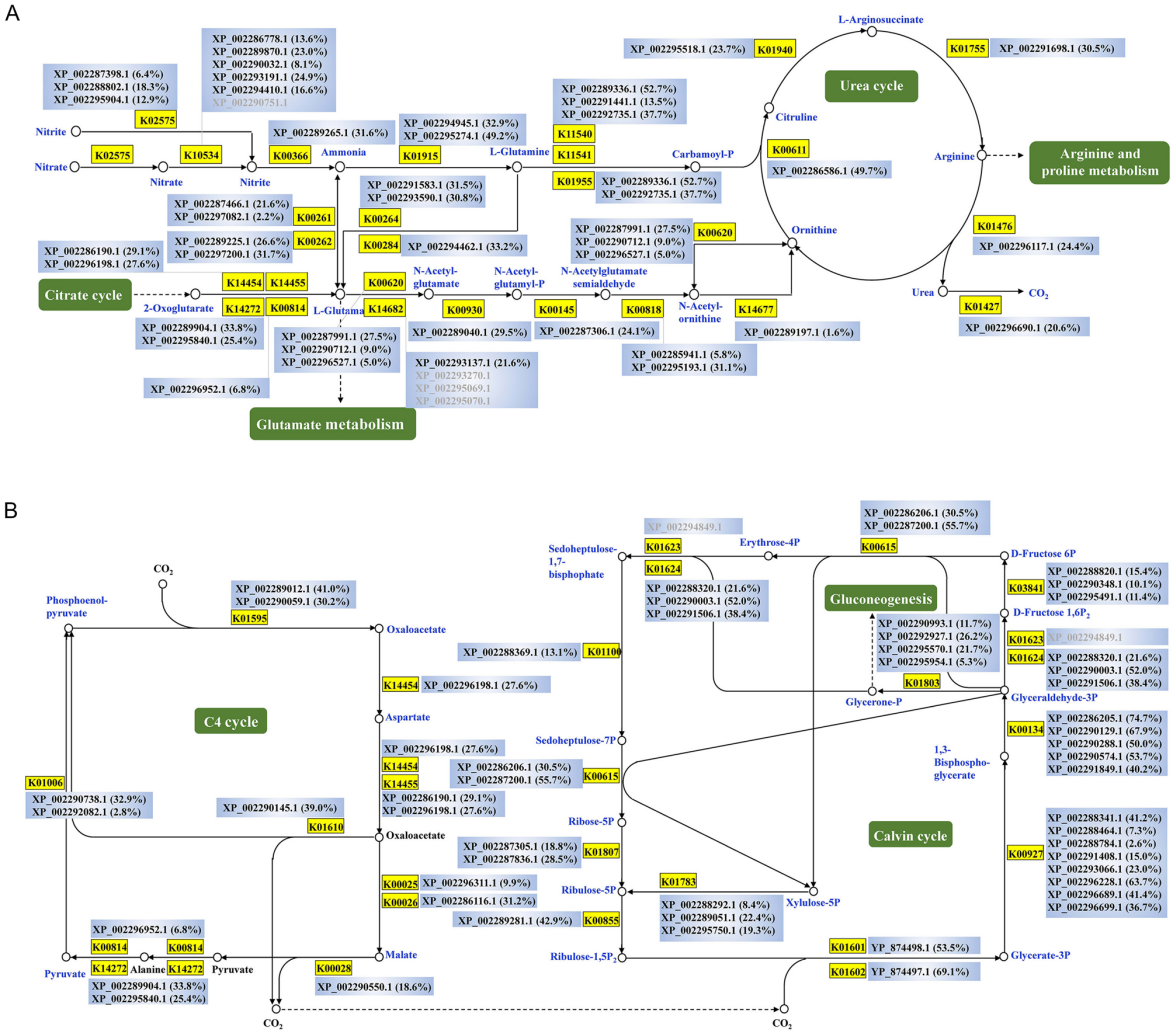
Identification of important biological processes and sequence coverage of proteins by the identified peptides. Brackets indicate sequence coverage. **A** Nitrogen metabolism and the urea cycle; **B** Carbon fixation

Most proteins associated with various biological processes were detected, while several important biological processes including nitrogen metabolism, urea cycle, and carbon fixation process were fully detected (Fig. 3). In the urea cycle, the sequence coverage of carbamoyl phosphate synthase (NCBI accession number XP_002289336.1) and ornithine carbamoyltransferase (XP_002286586.1) was 52.7% and 49.7%, respectively (Fig. 3A). The key enzymes involved in carbon fixation, ribulose bisphosphate carboxylase large and small chains, were identified with a sequence coverage of 53.5% and 69.1%, respectively (Fig. 3B).

### Identifying the novelty by proteogenomic analysis

**Fig. 4:**
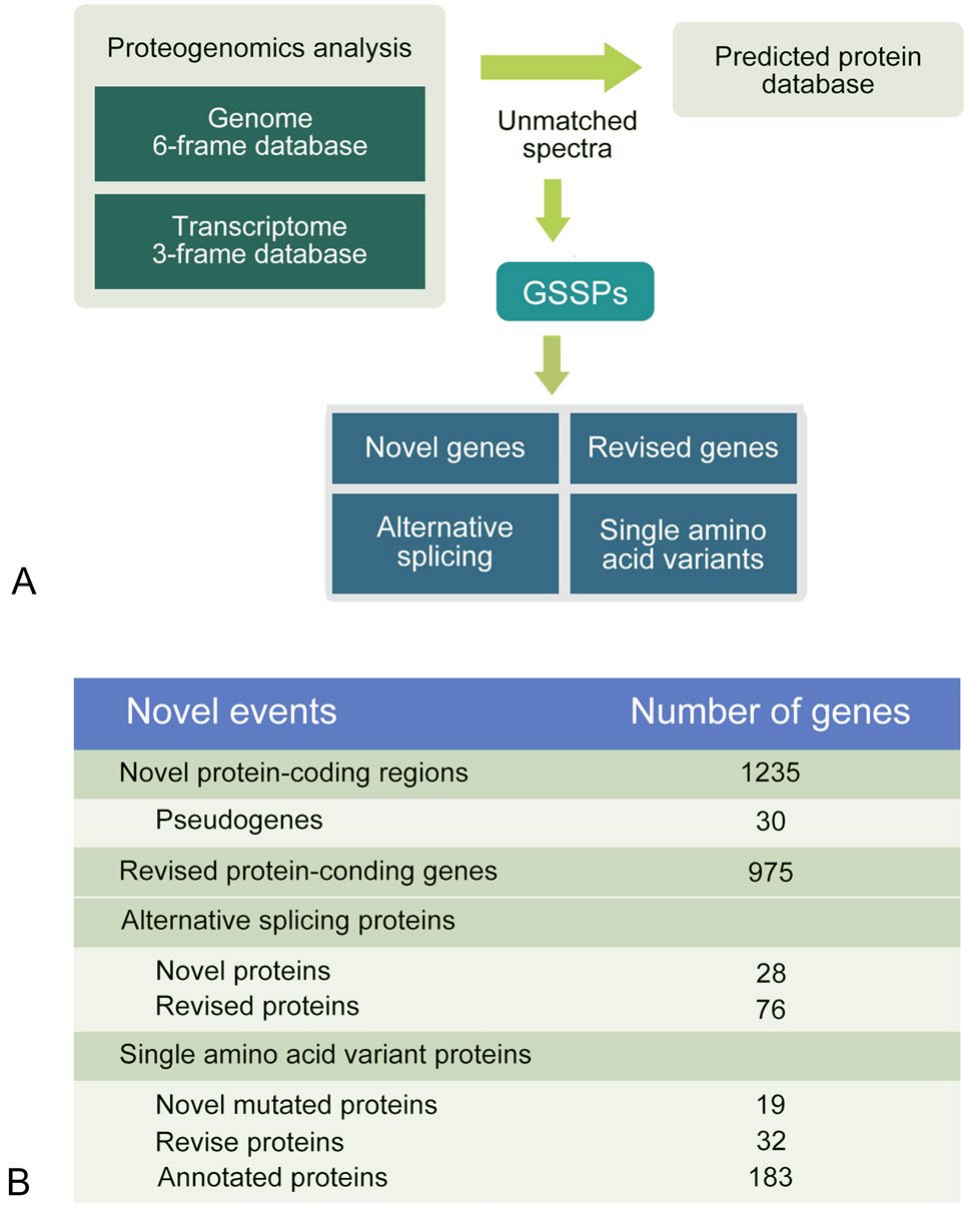
Proteogenomic analysis. **A** Workflow for proteogenomic analysis; **B** Summary of the results from proteogenomic analysis

To identify potential novelties in the *T. pseudonana* reference genome, a proteogenomic analysis was performed by a search using the MS data against the six-frame translation of genomic sequences and three-frame translation of RNA sequences (Fig. 4A). Genome search-specific peptides (GSSPs), the novel peptides that did not match currently predicted gene models, were extracted to predict novel genes. As a result, 1235 novel protein-coding genes with at least two unique GSSPs, including 30 predicted pseudogenes, were discovered (Fig. 4B and Supplementary Data Set 3). Our analysis also resulted in 975 gene models being corrected by at least two unique GSSPs (Fig. 4B and Supplementary Data Set 3). Furthermore, 104 splice variants, including 28 novel- and 76 revised alternative splicing proteins were discovered (Fig. 4B and Supplementary Data Set 3). Two hundred and thirty-four single amino acid variants, including 19 novel proteins, 32 revised proteins and 183 annotated proteins were also identified (Fig. 4B and Supplementary Data Set 3). The distribution of these identified novelties on different chromosomes is shown in Fig. 2.

**Fig. 5:**
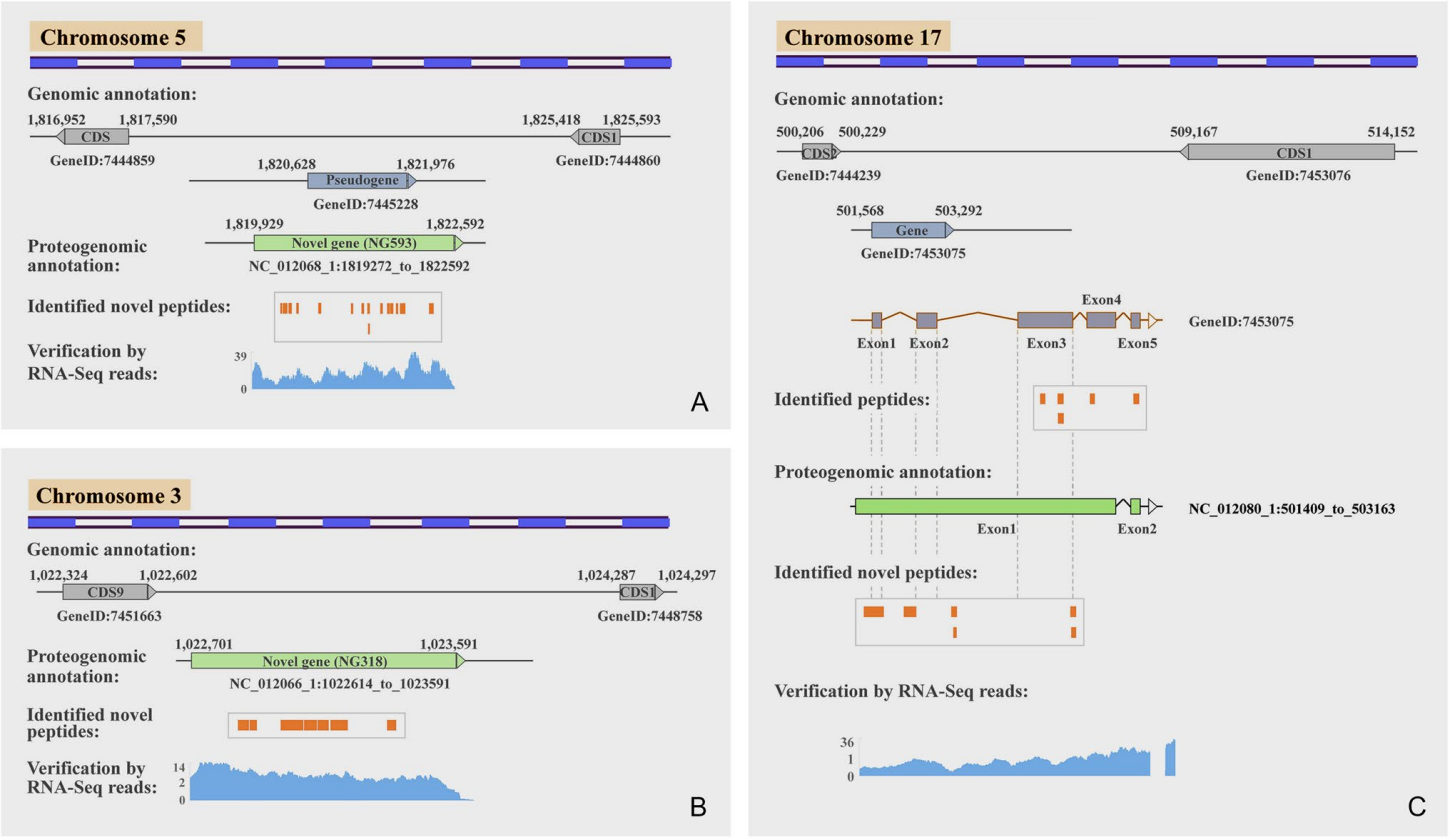
Identification of novel genes and revised gene models. **A** Novel peptides mapping to intergenic regions; **B** Novel peptides mapping to pseudogene regions; **C** Revision of a gene model by novel peptides

Figure 5A shows that a novel gene (NG593) annotated as phosphoric ester hydrolase was identified by mapping 16 unique novel peptides to the intergenic regions where a previously predicted pseudogene was located. The identified translation product of these presumed pseudogenes indicated that these genes can indeed encode proteins. Furthermore, a novel gene (NG318) annotated as calcium-dependent phospholipid binding was also identified by mapping seven unique novel peptides to the intergenic regions (Fig. 5B). The current RNA sequencing (RNA-seq) data further support the identification of these genes.

The MS data were also used to correct the gene models of already annotated genes. For example, Fig. 5C shows how the structure of a gene (GeneID: 7453075) was corrected by merging five exons into a single long one using a revised gene model. Five identified unique peptides mapped to exons 3–5 of this predicted gene, and seven unique novel peptides (i.e., peptides mapping to an intron) were also identified. Among them, three novel peptides were mapped to intron–exon 1 region, exon 1-intron region, and the intron region between exons 1 and 2, respectively; two novel peptides were mapped to the intron region between exons 2 and 3; and the remaining two unique novel peptides were mapped to the intron region between exons 3 and 4. The revised structures were also validated by RNA-seq data. These results indicated the existence of read-through between exon 1-exon 2, exon 2-exon 3 and exon 3-exon 4, and the extension of the 5′ terminal.

A group of novel splice sites was discovered using peptides spanning exon-exon boundaries (Supplementary Fig. S3A, B). A novel alternative splicing protein was identified by mapping 15 unique novel peptides and a splice junction peptide to intergenic regions (Supplementary Fig. S3A), while a revised alternative splicing protein was identified by mapping 15 unique novel peptides and a splice junction peptide to regions that overlapped an annotated gene (Supplementary Fig. S3B). The existence of novel splice sites was supported by RNA-seq data. In addition, many single amino acid variants were also identified. Supplementary Fig. S3C shows an annotated gene containing two mutant peptides, in which codon CCC (proline) was mutated to codon TCC (serine) and codon GAT (aspartate) was mutated to codon GTT (proline). As shown in Supplementary Fig. S3D, a novel gene mapping to a pseudogene region was also identified, in which a mutation of codon TTT to CTT resulted in a phenylalanine to leucine substitution.

### Functional annotation of novel genes

Among the 1235 novel genes, 776 were annotated with GO terms (Supplementary Fig. S4). According to this analysis, a large number of novel genes are involved in metabolic processes, cellular process, cellular metabolic processes, nitrogen compound metabolic processes, organic substance metabolic process and primary metabolic processes.

Many novel genes had the same biological function as pre-existing genes already in the genome, including many isozymes, but a subset of novel genes with novel biological functions was also discovered (Supplementary Data Set 2). For example, a plant cysteine oxidase (*CDO*) involved in hypotaurine biosynthesis, a nicotinate (nicotinamide) nucleotide adenylyltransferase (*NMNAT*) participating in nicotinamide adenine dinucleotide (*NAD*) biosynthesis, and several novel genes involved in vitamin biosynthesis and metabolism, including δ-24(24(1))-sterol reductase (*ERG4*), homogentisate phytyltransferase (*HPT*) and biotin-[acetyl-CoA-carboxylase] ligase (*birA*) were identified. A cytoplasmic adenylosuccinate synthetase involved in purine biosynthesis and a pseudouridylate synthase/pseudouridine kinase participating in uracil biosynthesis were also detected among the novel genes. Some proteins involved in carbohydrate and glycan metabolism, such as a 3-hexulose-6-phosphate synthase (*hxlA*) catalyzing the conversion between ribulose 5-phosphate and 3-hexulose 6-phosphate, class T phosphatidylinositol glycan (*PIGT*), and a polyprenol reductase involved in N-glycan biosynthesis were also found. In the carotenoid biosynthesis pathway, another phytoene dehydrogenase, phytoene desaturase (3,4-didehydrolycopene-forming, *AL1*) was identified. Furthermore, some novel genes involved in ribosome biosynthesis, ubiquitin system, protein palmitoylation, spliceosome and RNA modification were also identified.

### Sequence homology analysis of novel proteins

**Fig. 6:**
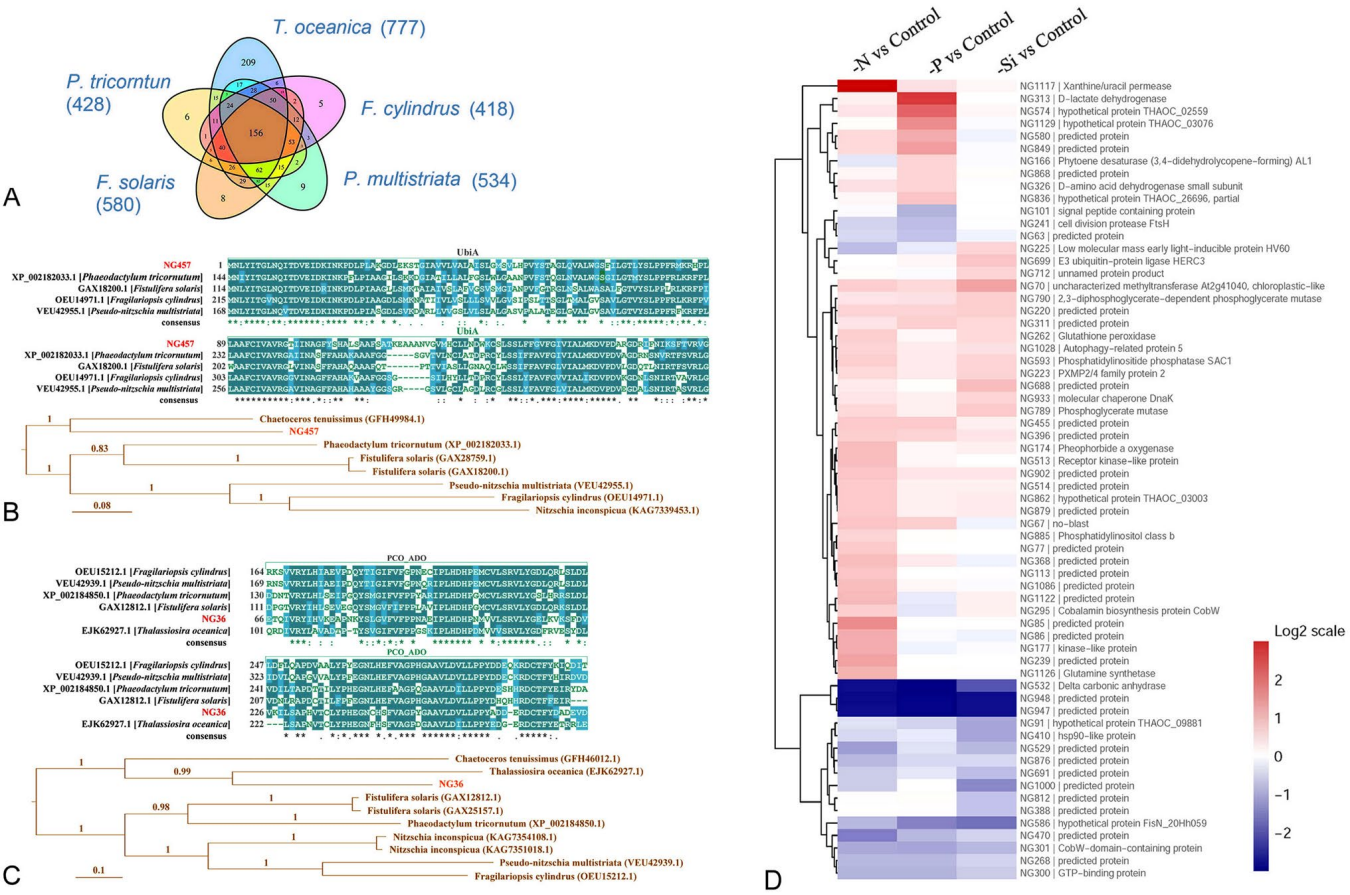
Sequence analysis and differential protein expression analysis of novel proteins. **A** shows the Venn diagram of the distribution of novel proteins with homology to proteins from the five diatoms (*Thalassiosira oceanica*, *Phaeodactylum tricornutum*, *Fistulifera solaris*, *Pseudo-nitzschia multistriata* and *Fragilariopsis cylindrus*). **B** and **C** show the sequence alignments for the domain of novel protein NG457 (**B**), NG36 (**C**) and the result of the phylogenetic analysis of homologous amino acid sequences from other diatom species. Numbers no the branches indicate posterior probability values and the scale bares indicate the number of expected changes per site along the branches. Accession numbers from NCBI for the analyzed amino acid sequences are also indicated. **D** shows the heatmap of novel gene expressions under different nutrient limitations

Sequence similarity comparison can be used to further verify the presence and function of the novel genes. To that end, a total of 1235 novel protein sequences were blasted against the NCBInr database, and 1019 of them were found to have a significant match with a protein in another species, including other diatoms (Supplementary Data Set 4). Among them, 922 novel proteins were found to be homologous to proteins from five other sequenced diatoms, *Thalassiosira oceanica*, *Phaeodactylum tricornutum*, *Fistulifera solaris*, *Pseudo-nitzschia multistriata* and *Fragilariopsis cylindrus*. Furthermore, homologous sequences for 777 novel proteins were found in the closest whole genome sequenced species *T. oceanica*, something that further strengthens the notion that these are functional protein-coding genes. The distribution of novel genes that are significantly similar to those in other diatoms is shown in Fig. 6A. An example of this synteny across diatom genomes, amino acid pairwise sequence alignments between the novel gene NG457 in *T. pseudonana* and orthologous regions from other diatom genomes shared > 53% identity. The UbiA domain was found in all protein sequences, suggesting that the biological function of NG457 is related to that of the UbiA prenyltransferase family. The multi-sequence alignment and phylogenetic tree also revealed that the novel protein NG457 is conserved and easily recognized in other diatom species (Fig. 6B). Analysis of sequence alignments and the phylogenetic tree of the novel protein NG36 suggested that this gene is also conserved in other diatoms, including the closest whole sequenced species *T. oceanica* (Fig. 6C).

### Validation of novel genes using quantitative proteomic data

Besides using RNA-seq data and sequence homology analysis for the validation of novel genes, expression of these novel genes was also verified by our previous quantitative proteomic data of *T. pseudonana* grown in nitrogen-, phosphate- and silicon-replete and -deficient conditions (Chen et al. 2018). A total of 1235 novel protein sequences were added to form a new protein sequence database, which will be used for protein re-identification. As a result, 531 novel proteins were detected and confirmed by re-identification (Supplementary Data Set 5). Among them, 64 novel proteins were differentially expressed among three nutrient-deficient cells compared with the nutrient-replete cells, including 38 novel proteins under nitrogen deficiency, 25 novel proteins under phosphate deficiency and 22 novel proteins under silicon deficiency (Fig. 6D and Supplementary Data Set 5). The re-identification of these novel genes and their differential expressions under different nutrient conditions further verifies the presence of these novel genes.

A novel δ-carbonic anhydrase was significantly downregulated among the three different nutrient-deficient cells (Fig. 6D). Under nitrogen-deficient conditions, a group of novel proteins, including a xanthine/uracil permease, a glutamine synthetase and a pheophorbide A oxygenase, were markedly upregulated, especially the xanthine/uracil permease which was upregulated by 7.81-fold. Under phosphate-deficient condition, a cell division protease (FtsH) was significantly downregulated, while a D-lactate dehydrogenase and a calcium/calmodulin-dependent protein kinase I were highly expressed; especially D-lactate dehydrogenase, which was 5.01-fold upregulated. These results indicate that the novel genes supplement a group of differentially expressed proteins, which provide more comprehensive evidence of the responses of *T. pseudonana* to ambient nitrogen, phosphate or silicon deficiency.

## Discussion

Much effort has been devoted to the genomic studies of marine diatoms and ten diatom genomes have to date been sequenced (Armbrust et al. 2004; Basu et al. 2017; Bhattacharjya et al. 2021; Bowler et al. 2008; Galachyants et al. 2019; Lommer et al. 2012; Mock et al. 2017; Ogura et al. 2018; Osuna-Cruz et al. 2020; Tanaka et al. 2015). However, a high-quality map of marine diatom proteomes is still lacking (Nunn et al. 2009; Yang et al. 2018). In this study, we identified 9526 predicted proteins from the model diatom *T. pseudonana*, which accounted for ~ 81% of the predicted protein-coding genes in the *T. pseudonana* genome. This is the most complete protein identification with the highest identified coverage in diatom proteomic studies reported so far. Such a high protein identification ratio indicates the success of developing a proteome map of *T. pseudonana* compared with draft proteome maps of other species (Kim et al. 2014; Yang et al. 2014, 2018). The expression of many important biological processes predicted in the *T. pseudonana* genome was fully detected in this study, such as the urea cycle, C4 photosynthesis, Calvin cycle, and nitrogen metabolism. The complete urea cycle was discovered in the diatom genomes (Armbrust et al. 2004; Allen et al. 2011) and showed that carbamoyl phosphate synthase catalyzes the first step of the reaction of the urea cycle, and utilizes glutamine rather than ammonia in this process. All these enzymes were detected in our study and the present results demonstrate that our pipeline was successful in obtaining a high-quality proteome map. Compared with the results obtained by integrating the separation of protein levels by gel electrophoresis and the fractionation of peptide level by HPLC, as well as multi-enzyme digestion (Kim et al. 2014; Yang et al. 2014, 2018), we applied a strategy that combined protein sequential extraction with protein enrichment, according to the protein solubility. Multiple search engines, including MSGF + , X!Tandem and Mascot, were used in our study and this provided higher sensitivity and specificity than any single search engine, as well as better peptide identification (Yang et al. 2018; Yu et al. 2010). Additionally, samples from different growth phases and nutrient-deficient conditions also contributed to protein identification. We therefore envisage that our study should provide a reliable strategy to develop proteome maps for other organisms.

Despite the high quality of the proteome data, ~ 2000 predicted protein-coding genes in the *T. pseudonana* genome were not identified. These genes may not encode proteins or may not be translated under the experimental conditions used here, or are rapidly degraded under normal or nonspecific conditions, or likely were hard to purify from cell extracts. Furthermore, some peptides might be lost in the analysis because of low abundance or low solubility due to hydrophobicity, thereby being disadvantaged by the protein extraction methods.

In this study, an integrated proteogenomic analysis was performed to improve the genome annotation of the marine diatom *T. pseudonana*. We identified 1235 novel genes, 975 revised genes, 104 splice variants and 234 single amino acid variants. We also revised 30 mis-annotated pseudogenes as protein-coding genes. Our findings highlighted the need for using high-resolution MS and integrated proteogenomic analysis to complement and improve genome annotation. The discovery of these novel genes further improves the genome annotation of *T. pseudonana*.

Pseudogenes have been considered as nonfunctional sequences in the genome for a long time. However, evidence from recent studies shows that many pseudogenes have some form of biological activity, which has attracted interest and concern in their accurate annotation and function (Pei et al. 2012). Kalyana-Sundaram et al. (Kalyana-Sundaram et al. 2012) systematically analyzed the pseudogene transcripts of RNA-seq data from 293 human samples representing 13 cancer and normal tissue types and found that the expression of pseudogenes is genome-wide and could be classified as either universally expressed, lineage specific or cancer specific. Recently, the translation of pseudogenes has been detected using proteogenomic analysis based on MS data. Kim et al. (Kim et al. 2014) identified 200 novel peptides encoded by 140 pseudogenes using proteogenomic analysis and found that translation of about half the pseudogenes was cell or tissue specific, while a minority were translated universally. In our study, of the 1235 novel protein-coding genes identified by the proteogenomic method, 30 were mapped to the regions of pseudogene sequences. The expression of these presumed pseudogenes was further confirmed by RNA-sequence data, and the products of two pseudogenes were significantly upregulated under nitrogen-deficient conditions in our quantitative proteome data. Our findings suggest that some protein-coding sequences have been mis-annotated as pseudogenes in the *T. pseudonana* genome and the accuracy of pseudogene annotation needs verification.

Noncoding RNAs (ncRNAs) are transcripts that do not encode proteins yet still play a role in gene expression and regulation (Guttman et al. 2013). However, we found a group of novel peptides mapping to protein sequences from long noncoding RNAs (lncRNAs) annotated in transcriptomes that have been identified in human proteogenomic data (Kim et al. 2014). A previous study has also shown that lncRNAs may encode functional minipeptides (Anderson et al. 2015). In the diatom *P. tricornutum*, 64 lncRNAs belong to 56 novel genes identified by proteomic data, thus confirming that these transcripts are incorrectly annotated as lncRNAs (Yang et al. 2018). Our proteogenomic data can thus provide a data source for verifying the accuracy of ncRNAs of *T. pseudonana* in future.

Many novel genes provide the same biological functions as previously predicted genes, whereas others show novel biological functions that have not been annotated previously. Homogentisate phytyltransferase (ubiA domain) catalyzes the first committed step of tocopherol biosynthesis in all photosynthetic organisms. A novel gene NG457 predicted to encode homogentisate phytyltransferase was discovered in this study, which completed the tocopherol biosynthesis pathway. A previous study has shown that several microalgal species can synthesize taurine, starting from cysteine or cysteamine (Tevatia et al. 2015). Cysteamine is the end product of coenzyme A degradation. In this pathway, hypotaurine can be produced via oxidation of cysteamine, which is catalyzed by ADO. Hypotaurine can be converted into taurine by spontaneous conversion or a putative hypotaurine dehydrogenase (Tevatia et al. 2015; Vitvitsky et al. 2011). In the present study, the identification of the gene NG36 with the PCO_ADO domain complemented the taurine biosynthesis process. Phytoene dehydrogenases are the key rate-limiting enzymes in the carotenoid biosynthesis pathway, which catalyze the production of ζ-carotene, neurosporene, lycopene, 3,4-didehydrolycopene, 3,4,3′,4′-tetradehydrolycopene or 3,4-didehydroneurosporene. The type of dehydrogenase determines the type of product. Genes encoding phytoene desaturase (PDS), ζ-carotene isomerase (Z-ISO), δ-carotene desaturase (ZDS) and cis-carotene isomerase (CrtISO) are found in the *T. pseudonana* genome (Bertrand 2010; Takaichi 2011), producing ζ-carotene, neurosporene and lycopene. Here, we identified the novel gene NG1119 that encodes another phytoene dehydrogenase, AL1, which is involved in production of 3,4-didehydrolycopene, and which further complements the carotenoid biosynthesis pathway in diatoms. Additionally, other pathways, such as vitamin biosynthesis and metabolism, glycans metabolism, ribosome biosynthesis, ubiquitin system, protein palmitoylation, spliceosome and RNA modification, were also supplemented by novel genes with novel biological functions. These findings further demonstrate the necessity of identifying novel genes through proteogenomic analysis to expand future diatom studies.

In summary, we have developed a relatively comprehensive proteome map of *T. pseudonana* that contains 9526 proteins, accounting for ~ 81% of the predicted protein-coding genes. Most protein-coding genes involved in many important biological processes were completely detected, such as the urea cycle, C4 photosynthesis, Calvin cycle, and nitrogen metabolism. Furthermore, we applied a comprehensive proteogenomic analysis strategy to complement the genome annotation. This approach revealed 1235 novel protein-coding genes that are missing in the current *T. pseudonana* genome annotation, which updated the protein database of *T. pseudonana* and further complement the proteome catalog. This study provides a better reference for developing proteomic landscapes of other whole genome sequenced diatoms, and constitutes a valuable resource for the diatom research, marine ecology and biogeochemistry communities.

## Materials and methods

### Organism and culture conditions

*T. pseudonana* CCMP 1335 was maintained in f/2 medium at 20 ℃ in a 14:10 h light: dark photoperiod at a light intensity of approximately 100 μmol photons m^−2^ s^−1^ provided by fluorescent lamps. Before the experiment, *T. pseudonana* cells were treated with an antibiotic cocktail to eliminate bacteria from the culture medium. After that, the cells were washed three times with autoclaved seawater to remove remnant antibiotics and inoculated into fresh f/2 medium (Guillard and Ryther 1962). After 3 days growth, the viable cells at the exponential growth phase were transferred into three 5-L flasks each containing 4 L fresh f/2 medium. Cells at the exponential, stationary and decline phases were then harvested for the proteomic analysis.

### Protein extraction and enrichment

To mitigate protein loss caused by preferences of different protein extraction methods, we applied two sequential extraction methods to the harvested cells. The first method used a Sequential Extraction Kit (Cat No. 1632100, Bio-Rad) to extract proteins using reagents in three steps according to protein solubility. An appropriate amount of tributyl-phosphine was added to the reagents of the kit in advance as the reducing agent. Cell pellets were first lysed in Reagent 1 containing 40 mmol/L Tris with ultrasonic disruption. The supernatant was collected as Lysate 1 by centrifugation at 20,000*g* for 10 min at 4 °C. The precipitate was then washed twice with Reagent 1 and the washing supernatant was collected and combined with Lysate 1. The precipitate was further sequentially lysed in Reagent 2 (8 mol/L urea, 4% CHAPS, 40 mmol/L Tris, 0.2% Bio-Lyte 3/10 ampholyte) and Reagent 3 (5 mol/L urea, 2 mol/L thiourea, 2% CHAPS, 2% SB 3–10, 40 mmol/L Tris, 0.2% Bio-Lyte 3/10 ampholyte) with ultrasonic disruption, and Lysates 2 and 3 were collected. Proteins from the three lysates were precipitated using 20% (w/v) trichloroacetic acid/acetone solution at − 20 ℃ overnight, centrifuged at 20,000*g* for 20 min at 4 ℃, and washed twice with cold acetone. Finally, the precipitate was redissolved in rehydration buffer containing 7 mol/L urea, 2 mol/L thiourea, 2% SDS and 40 mmol/L Tris, and the protein solution was collected by centrifugation at 20,000*g* for 10 min at 16 ℃. The second protein extraction method was conducted in two steps. Cells were first extracted in the regent containing 40 mmol/L Tris with ultrasonic disruption as a first step, then the lysate was treated as described in the first method. The residual precipitate was further extracted using TRIzol reagent as described previously (Wang et al. 2012). Finally, the protein precipitate was redissolved in the rehydration buffer as described above.

In the two protein extraction methods, the Tris reagent extracted hydrophilic proteins, such as cytosolic proteins, Reagent 2 extracted relatively water-insoluble proteins, Reagent 3 extracted hydrophobic proteins, while TRIzol reagent extracted both hydrophilic and hydrophobic proteins. The amount of extracted protein was low in the second and third steps of both sequential extraction methods. To enrich more water-insoluble or hydrophobic proteins, we applied an enrichment strategy. In brief, more algal cells were extracted to obtain enough proteins in the second and third steps, and subsequently equal amounts of protein from each step of the different extraction methods were mixed into one sample. The extractions were performed in three biological replicates which were subsequently pooled. Protein concentration was measured using the 2-D Quant kit (GE Healthcare).

### Peptide fractionation and LC–MS/MS analysis

A total of 100 μg protein from each sample was digested with Trypsin Gold (Promega) in 0.5 mol/L TEAB in a 10-kDa ultrafiltration device (Millipore) after being reduced and alkylated. The peptides were desalted using Strata X column (Phenomenex) and dried in a vacuum centrifuge. The dried peptides were reconstituted with 2 mL buffer A (5% acetonitrile, pH 9.8) and fractionated with a 4.6 mm × 250 mm Gemini C18 column (Phenomenex) on an LC-20AB HPLC system (Shimadzu). Peptides were separated at a rate of 1 mL/min with a gradient of 5% buffer B (95% acetonitrile, pH 9.8) for 10 min, 5–35% buffer B for 40 min, 35–95% buffer B for 1 min, maintained in buffer B for 3 min and then returned to 5%. The 20 fractions were collected based on the elution peaks monitored at 214 nm wavelength. Peptides from each fraction were reconstituted in buffer C (2% acetonitrile and 0.1% formic acid) after being dried, then separated on an LC-20AD nano-HPLC (Shimadzu). Peptides were eluted at a flow rate of 300 nl/min with a gradient of 5% buffer D (98% acetonitrile and 0.1% formic acid) for 8 min, 8–35% buffer D for 35 min, 35–60% buffer D for 5 min, 60–80% buffer D for 2 min, and maintained in 80% buffer D for 5 min and then returned to 5%. Peptide separation was followed by MS/MS Q-Exactive (Thermo Fisher Scientific) after nanoelectrospray ionization. The MS and MS/MS scans were, respectively, operated at a resolution of 70,000 and 17,500. The 20 most abundant precursor ions above a threshold intensity of 10,000 with a 2 + to 7 + charge state was selected for MS/MS using high-energy collision dissociation. The dynamic exclusion duration was set to 15 s.

### Proteogenomic databases and peptide identification

The latest revision of the *T. pseudonana* protein sequences and genome sequences, which was improved in 2008 (Bowler et al. 2008), were downloaded from the NCBI website (https://www.ncbi.nlm.nih.gov/genome/?term=Thalassiosira+pseudonana). The *T. pseudonana* reference genome sequence is provided by NCBI, and the same version is also available on the Joint Genome Institute (https://genome.jgi.doe.gov/portal/Thaps3/Thaps3.download.html; https://genome.jgi.doe.gov/portal/Thaps3_bd/Thaps3_bd.download.html). Organelle annotations were also added for identification (Armbrust et al. 2004; Oudot-Le Secq et al. 2007). RNA-seq raw-read data were retrieved from the GEO database (http://www.ncbi.nlm.nih.gov/geo) (Accession no. GSE75460) (Smith et al. 2016). The transcripts were assembled into long transcripts using Trinity ve.2.0.6 after the low-quality reads were filtered out with Trimmomatic v.0.32 program (Grabherr et al. 2011). Subsequently, sequences < 50 base pairs in length were removed, after which a six-frame-translated genome database and a three-frame-translated transcriptome database were created.

Raw MS data were converted to “mgf” format using the MSConvert tool in the ProteoWizard software v.3.0.4416. Three different search algorithms, MSGF + (Kim and Pevzner 2014), X!Tandem (Craig and Beavis 2004), and Mascot (Perkins et al. 1999), were then used to analyze the acquired data. The main search parameters were set as follows: two maximum missed cleavage sites for trypsin, the precursor ion mass tolerance of 10 ppm and the fragment ion mass tolerance of 0.05 Da; a fixed modification of carbamidomethylation (Cys); and dynamic modifications of oxidation (Met), deamidation (Asn/Gln) and acetylation (protein N-terminal). MS spectra mapped to different sequences in different searches were filtered off. A more stringent FDR filtering strategy was used to evaluate peptide identification (Wen et al. 2016; Yang et al. 2018). For all proteins identified by one unique peptide, all MS/MS spectra of unique peptides were manually checked using a method as previous described by Macek et al. (2008). After removing the identified peptides mapping to the predicted amino acid sequence database by BLASTP, the GSSPs were obtained. Identified proteins of *T. pseudonana* under different nutrient conditions in our previous quantitative proteomic study (Chen et al. 2018) were also added to complement the complete proteome of *T. pseudonana*.

### Identification of novel genes, revised genes, alternative splicing and single amino acid variants

Novel genes were identified as the open reading frames (ORFs) mapped to non-protein-coding regions in the genome. Novel proteins had to contain at least two unique GSSPs. When the ORFs partially overlapped with a predicted gene or exon, they were used for revising gene models. The remaining GSSPs were then used to identify alternative splicing and single amino acid variants. At most, two nonsynonymous variants within a GSSP were allowed for the identification of single amino acid variant. The details of the identification protocol were described in a previous study (Yang et al. 2018). In addition, RNA-seq reads were mapped to the genome using STAR v.2.5.2a after filtering low-quality reads. Subsequently, the target regions were examined via the visualization tool of IGV v.2.5.0 and were verified with no more than three mismatches in each sample.

### Annotation of genes

All predicted gene models were annotated by blasting them to the SwissProt/UniProt databases, and by querying the KOG, GO and the KEGG databases. The subcellular localization of all predicted proteins was analyzed using the CELLO web tool (Yu et al. 2004). The location of all predicted genes and identified proteins in the genome of *T. pseudonana* were represented using Circos software (Krzywinski et al. 2009). The predicted biological function of identified novel proteins was annotated by GO terms and KEGG database.

### Sequence analysis of novel proteins

Prediction of domains of target novel proteins was analyzed using profile hidden Markov Models in online HMMER (https://www.ebi.ac.uk/Tools/hmmer/). The on-line version of Blastp (https://blast.ncbi.nlm.nih.gov/Blast.cgi?PROGRAM=blastp) was used for retrieving homologous sequences from other species. Sequence alignments were conducted using Clustal X v2.1 and the output files were processed using the online BOXSHASE sever (v3.21) to visualize the results.

A phylogenetic analysis was conducted, where the amino acid sequences of the novel genes NG36 and NG457 were used as query sequences on the NCBI nr database. The search was restricted to only diatom species (taxid: 2836) and identified homologous sequences were aligned using mafft v7.490 and the linsi setting. The two multi-sequence alignments where then analyzed separately using MrBayes v3.2.6 for 1′000′000 generations, sampling trees every 1′000 generations and using the mixed evolutionary model, after which the analyses were deemed to have converged. The first 25% of the trees were disregarded and the remaining tree samples were summarized in a majority consensus tree. The trees where manually rooted based on information from the NCBI taxonomy database (Fig. 6).

### Validation of novel genes in the quantitative proteomic data

Raw data from our previous iTRAQ-based quantitative proteome of *T. pseudonana* (Chen et al. 2018) was used for protein re-identification using Mascot v.2.3.02 (Matrix Science, London, U.K.). Eight peptide samples (two biological replicates for each sample) including nutrient-replete, N-deficient, P-deficient and Si-deficient samples were labeled with different iTRAQ tags (113-119 and 121). A new *T. pseudonana* protein database was created for MS/MS search by adding the 1235 novel-protein sequences from the proteogenomics analysis, then all the peak lists were searched against this more complete database. Trypsin was used as protease with two maximum missed cleavages. The precursor ion mass tolerance was set as 10 ppm and the fragment ion mass tolerance was set as 0.05 Da. The fixed modification was set as Carbamidomethyl (C), iTRAQ 8plex (N-term) and iTRAQ 8plex (K), while the variable modification was set as Oxidation (M) and iTRAQ 8plex (Y). After FDR filtering, identified novel proteins were selected for further analysis. Novel proteins identified with at least two unique peptides were selected for quantitation. Differentially expressed novel proteins were defined with the criteria of mean fold change > 1.5 or < 0.67, and *P* < 0.05.

## Supplementary Information

Below is the link to the electronic supplementary material.

Supplementary file1 (DOCX 1431 KB)

Supplementary file2 (XLSX 56959 KB)

Supplementary file3 (XLSX 1214 KB)

Supplementary file3 (XLSX 2241 KB)

Supplementary file3 (XLSX 91 KB)

Supplementary file3 (XLSX 77 KB)

## Data Availability

The raw mass-spectrometry data have been deposited to the ProteomeXchange Consortium via the PRIDE partner repository (http://www.ebi.ac.uk/pride) (Perez-Riverol et al. 2019) with the dataset identifier PXD027392.
