# Supplementary material for: Improving the genome and proteome annotations of the marine model diatom Thalassiosira pseudonana using a proteogenomics strategy
Source: Mar Life Sci Technol. 2023 Feb 3;5(1):102–15. doi: 10.1007/s42995-022-00161-y (PMC10077189; doi:10.1007/s42995-022-00161-y)
Supplement: Supplementary file 1 — Supplementary file1 (DOCX 1431 KB) [file 42995_2022_161_MOESM1_ESM.docx]

Improving genome and proteome annotations of the marine model diatom *Thalassiosira pseudonana* using a proteogenomics strategy

Xiao-Huang Chen^1,2,4#^, Ming-Kun Yang^3#^, Yuan-Yuan Li^1^, Zhang-Xian Xie^1,2^, Shu-Feng Zhang^1,2^, Mats Töpel^5,6^,

Shady A. Amin^7^, Lin Lin^1,2^, Feng Ge^3*^, Da-Zhi Wang^1,2*^

^1^State Key Laboratory of Marine Environmental Science/College of the Environment and Ecology, Xiamen University, Xiamen 361005, China

^2^Southern Marine Science and Engineering Guangdong Laboratory (Zhuhai), Sun Yat-Sen University, Zhuhai, 519082, China

^3^Key Laboratory of Algal Biology, Institute of Hydrobiology, Chinese Academy of Sciences, Wuhan 430072, China

^4^College of Chemistry, Chemical Engineering and Environment, Minnan Normal University, Zhangzhou, 363000, China

^5^Department of Marine Sciences, University of Gothenburg, Box 461, 40530 Gothenburg, Sweden

^6^IVL - Swedish Environmental Research Institute, Gothenburg, Box 53021, 40014 Göteborg, Sweden

^7^New York University Abu Dhabi, Saadiyat Island, Abu Dhabi 129188, United Arab Emirates

#X.H.C and M.K.Y. contributed equally to this work

*Address correspondence to the following E-mails: gefeng@ihb.ac.cn or dzwang@xmu.edu.cn

**Supplementary Figures**

**Supplementary Fig. S1** Distribution of peptide number and sequence coverage of *Thalassiosira pseudonana* genome by mass spectrometry (MS) data. **A** Venn diagram showing the relative contribution of the peptide number identified at different growth phases. **B** Venn diagram showing the relative contribution of the peptide number identified using different search engines. **C** Distribution of unique peptide in the identified proteins based on peptide counts. **D** Sequence coverage of proteins by the identified peptides. **E** Identification of glyceraldehyde-3-phosphate dehydrogenase precursor (XP_002286205.1) with 75% sequence coverage and a representative MS/MS spectrum of peptide AVTKVIPSLQGK matching this protein.

**Supplementary Fig. S2** Annotation overview of function and subcellular localization of predicted genes and identified proteins. **A** Functional annotations; **B** Predicted subcellular localization.

**Supplementary Fig. S3** Identification of novel introns and single amino acid variants. **A** A novel intron of a novel gene by a splice peptide; **B** A novel intron of an annotated gene by a splice peptide; **C** Single amino acid variants of an annotated gene by mutated peptides; **D** A single amino acid variant of a novel gene by a mutated peptide.

**Supplementary Fig. S4** Gene Ontology (GO) functional annotation of novel genes.

**Supplementary Data Sets**

**Supplementary Data Set 1A**. List of PSMs identified by MSGF+ search engine.

**Supplementary Data Set 1B**. List of PSMs identified by X!Tandem search engine.

**Supplementary Data Set 1C**. List of PSMs identified by Mascot search engine.

**Supplementary Data Set 2A**. Functional annotation of all identified proteins in this study.

**Supplementaryl Data Set 2B**. List of non-identified proteins in this study.

**Supplementary Data Set 3A**. List of novel protein-coding genes.

**Supplementary Data Set 3B**. List of novel protein-coding genes previously annotated as pseudogenes in the genome.

**Supplementary Data Set 3C**. Revision of annotated gene models by revised genes.

**Supplementary Data Set 3D**. List of novel alternative splicing protein coding genes.

**Supplementary Data Set 3E**. List of revised alternative splicing protein coding genes.

**Supplementary Data Set 3F**. List of novel single amino acid variants.

**Supplementary Data Set 3G**. List of revised single amino acid variants.

**Supplementary Data Set 3H**. List of single amino acid variants from annotated proteins.

**Supplementary Data Set 4**. Results of sequence homology analysis of novel proteins.

**Supplementary Data Set 5A**. Novel proteins identified in nutrient-deficient samples.

**Supplementary Data Set 5B**. Differentially expressed novel proteins identified in nutrient-deficient samples.


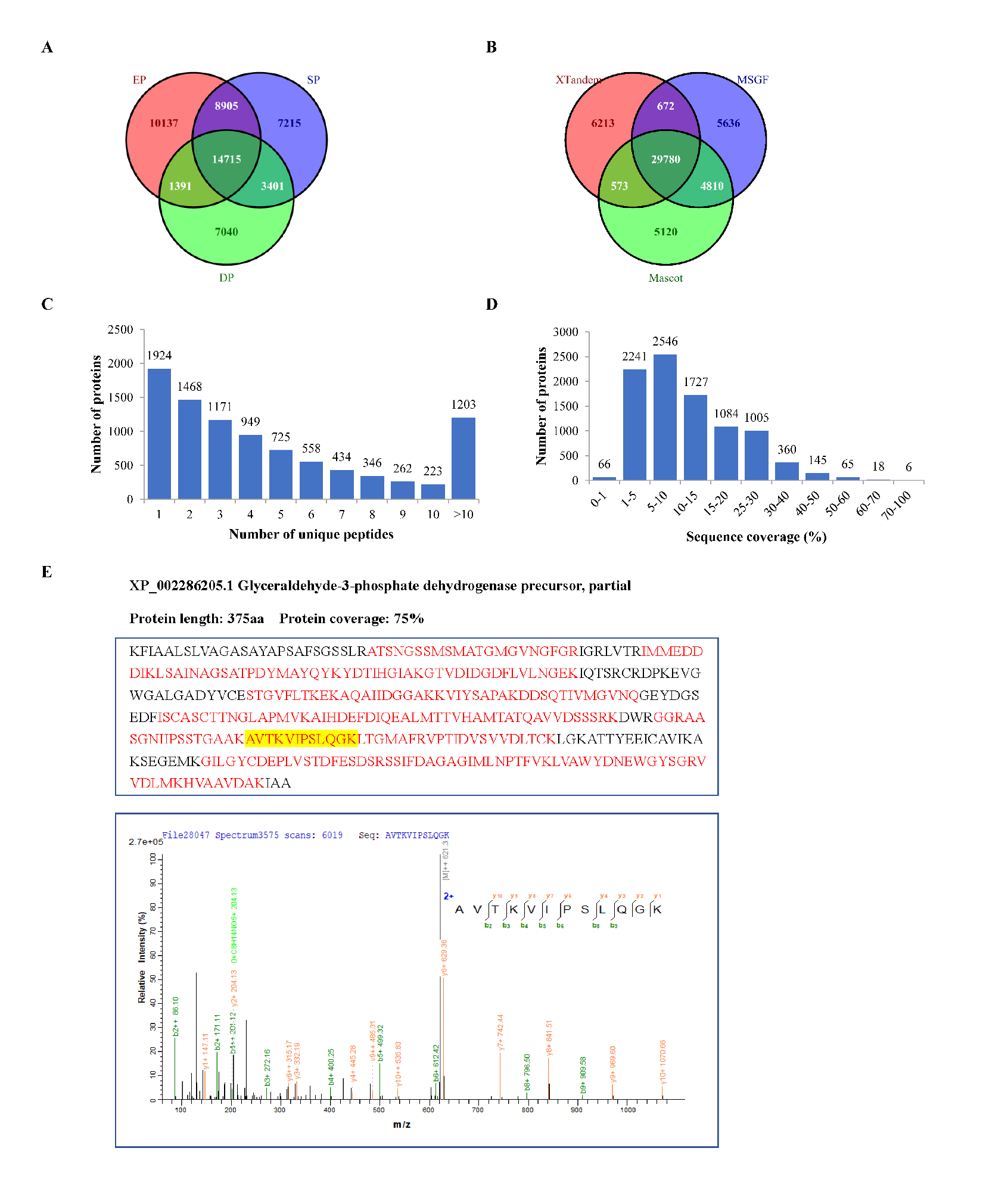


**Fig. S1** Distribution of peptide number and sequence coverage of the *Thalassiosira pseudonana* genome by mass spectrometry (MS) data. **A** Venn diagram showing the relative contribution of the peptide number identified at different growth phases. **B** Venn diagram showing the relative contribution of the peptide number identified using different search engines. **C** Distribution of unique peptide in the identified proteins based on peptide counts. **D** Sequence coverage of proteins by the identified peptides. **E** Identification of glyceraldehyde-3-phosphate dehydrogenase precursor (XP_002286205.1) with 75% sequence coverage and a representative MS/MS spectrum of peptide AVTKVIPSLQGK matching this protein.


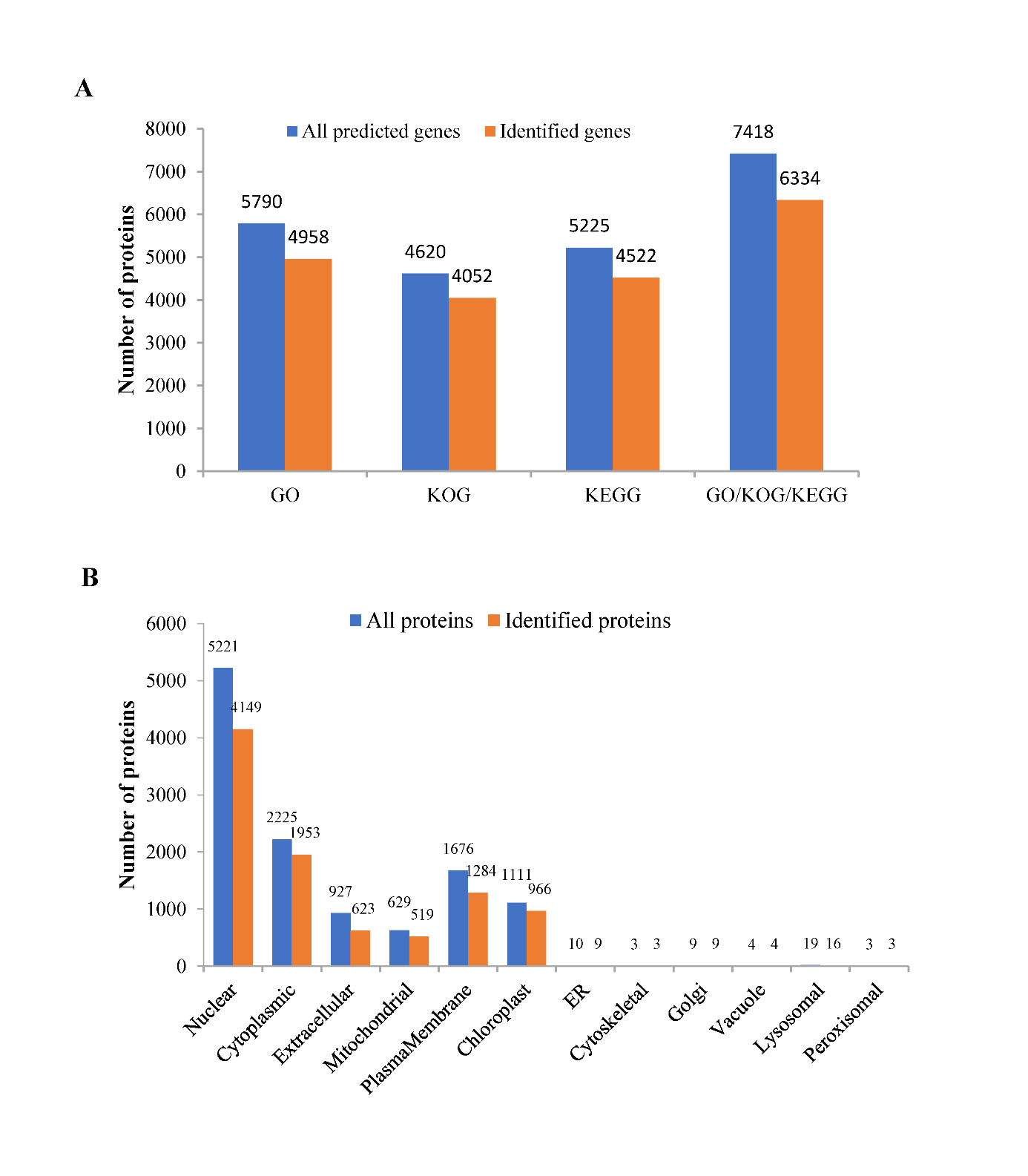


**Fig. S2** Annotation overview of function and subcellular localization of predicted genes and identified proteins. **A** Functional annotations; **B** Predicted subcellular localization.


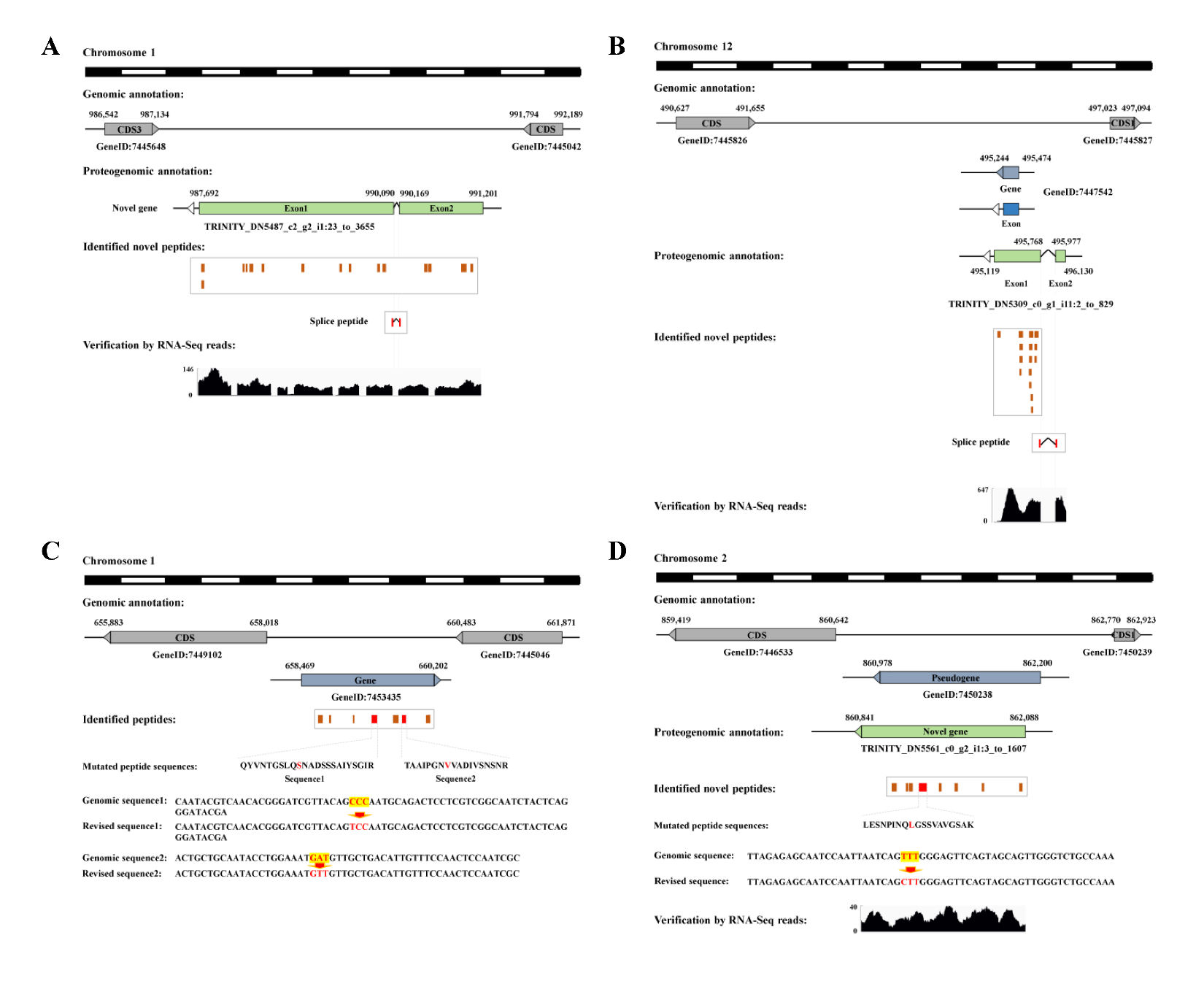


**Fig. S3** Identification of novel introns and single amino acid variants. **A** a novel intron of a novel gene by a splice peptide; **B** a novel intron of an annotated gene by a splice peptide; **C** single amino acid variants of an annotated gene by mutated peptides; **D** single amino acid variant of a novel gene by a mutated peptide.


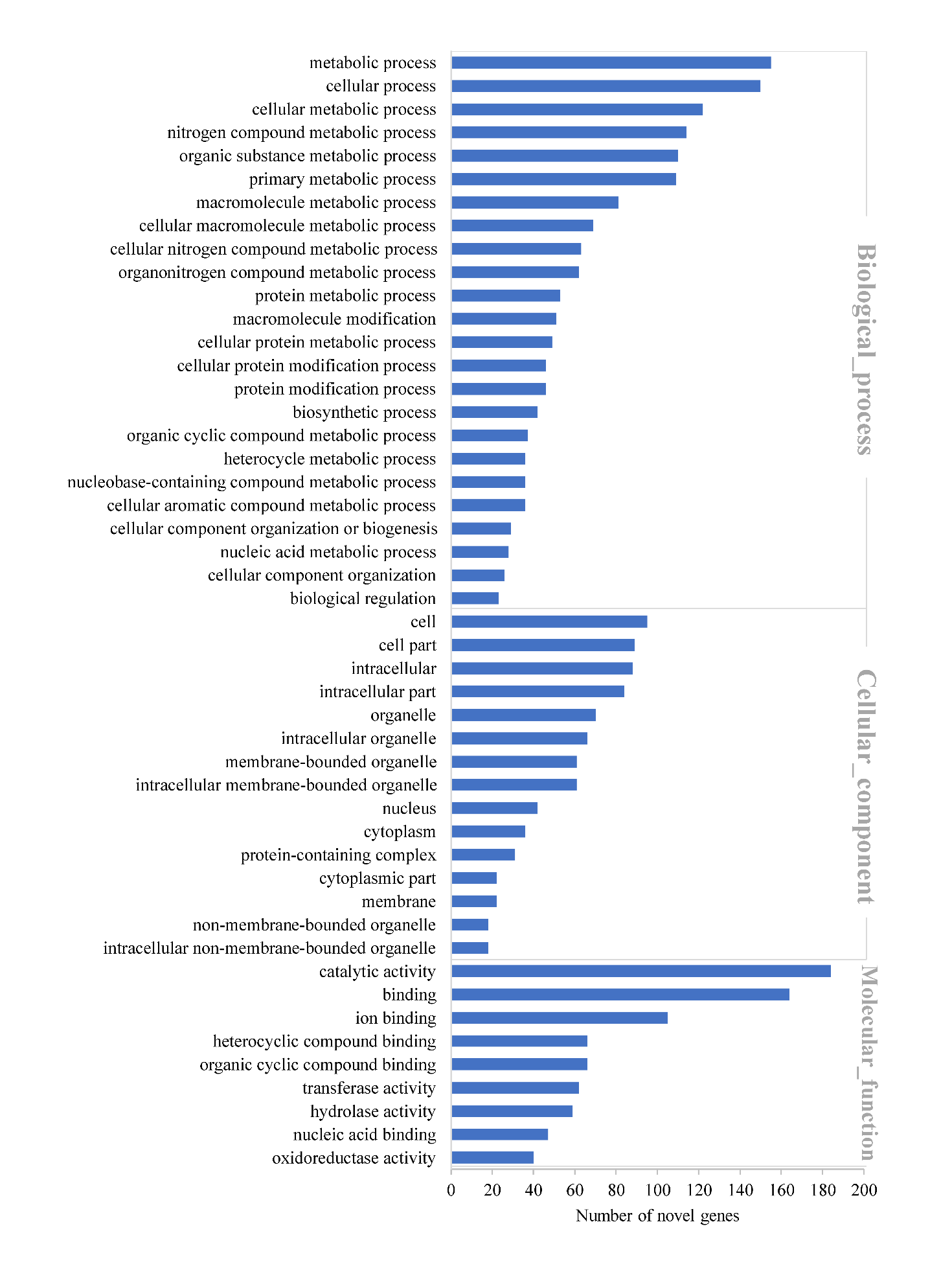


**Fig. S4** Gene Ontology (GO) functional annotation of novel genes.
